# Segmentation of Online Ferrograph Images with Strong Interference Based on Uniform Discrete Curvelet Transformation†

**DOI:** 10.3390/s19071546

**Published:** 2019-03-30

**Authors:** Leng Han, Song Feng, Guang Qiu, Jiufei Luo, Hong Xiao, Junhong Mao

**Affiliations:** 1 School of Advanced Manufacture Chongqing University of Posts and Telecommunications, Chongqing 400065, China; hanleng@cqupt.edu.cn (L.H.); qiuguangxq@163.com (G.Q.); jiufluo@gmail.com (J.L.); xiaohong@cqupt.edu.cn (H.X.); 2 Key Laboratory of Education Ministry for Modern Design and Rotor-Bearing Systems, Xi’an Jiaotong University, Xi’an 710049, China; jhmao@xjtu.edu.cn

**Keywords:** ferrography, uniform discrete curvelet transformation, binarization

## Abstract

Through real-time acquisition of the visual characteristics of wear debris in lube oil, an on-line visual ferrograph (OLVF) achieves online monitoring of equipment wear in practice. However, since a large number of bubbles can exist in lube oil and appear as a dynamically changing interference shadow in OLVF ferrograms, traditional algorithms may easily misidentify the interference shadow as wear debris, resulting in a large error in the extracted wear debris characteristic. Based on this possibility, a jam-proof uniform discrete curvelet transformation (UDCT)-based method for the binarization of wear debris images was proposed. Through multiscale analysis of the OLVF ferrograms using UDCT and nonlinear transformation of UDCT coefficients, low-frequency suppression and high-frequency denoising of wear debris images were conducted. Then, the Otsu algorithm was used to achieve binarization of wear debris images under strong interference influence.

## 1. Introduction

The wear debris in lube oil are closely related to the wear state of the machines. The size and form of the debris can reflect the wear level and reveal the content and temperature rise of the particular material. Therefore, oil debris analysis has become an essential condition monitoring technique that is utilized to diagnose wear and serve as an early warning system. To realize online wear monitoring, many particle detection methods have developed rapidly [1]. The electromagnetic wear detection sensor and image wear detection sensor are commonly used in online debris monitoring. When metal particles pass through the detection coil, they change the inductance of the coil or the magnetic flux through the coil such that the electromagnetic debris sensor can detect the inductive voltage of the coil, which can be measured in real time to allow for abrasive particle monitoring [2,3,4,5]. For the image wear detection sensor, detection is mainly based on transmission imaging [6] and reflection imaging [7] with a high detection accuracy of up to 5 microns.

Online visual ferrography (OLVF) is an important image wear detection sensor. Separating ferromagnetic wear debris from lube oil using a high-gradient magnetic field, obtaining ferrograms using an image sensor, and extracting wear debris information, such as size, shape, and concentration, by ferrogram, online visual ferrography (OLVF) achieves online equipment wear monitoring. Compared with online monitoring technology based on inductive sensors, ultrasonic transducers and electrostatic sensors [8], OLVF can directly obtain visual information of the wear debris, reflecting wear conditions and realizing the monitoring of wear debris with a large size range, which has been applied in the evaluation of the anti-wear properties of lube oils, in automotive engine bench tests, and in gear wear monitoring [9,10,11].

Wear debris image processing, as an important prerequisite for equipment wear monitoring, and has been widely studied in recent years. Wu et al. [12] studied the preprocessing method of wear debris images, conducted comparative analysis of the grayscale processing effects of wear debris images through different-colored spaces, discussed the application effects of the background subtraction method and automatic threshold value method in the segmentation of the wear debris image, and proposed a suitable quantitative description method for wear debris images. Roylance et al. [13] studied the binarization, denoising and edge tracking of wear debris images, and extracted the features of wear debris, such as size, shape, and edge details. Zhan et al. [14] studied preprocessing techniques for wear debris images, such as image smoothing, image filtering, and image binarization, and processed the actual images using the blank convolution method, which effectively reduced noise and simplified the data processing process. Hu et al. [15] improved image quality using two filtering methods, for the convenience of subsequent object segmentation and extraction. In addition, Stachowiak et al. [16] studied the application of wavelet analysis in the automatic debris classification system, which could achieve a more reliable diagnosis of mechanical health. Wang et al. [17], while using the combined watershed and improved ant colony algorithm to segment the particle images, demonstrated the possibility of accurate image segmentation, including large abnormal particles and a sedimentary chain. Afterwards, a nonreference evaluation method for the edge detection of wear particles in ferrographic images was proposed by Wang et al. [18]; the wear particles obtained by this method are similar to those observed by the human eye, meaning that the evaluation results are simultaneously objective and reasonable. Wu et al. [19] studied a method to detect dynamic particles, obtaining three-dimensional abrasive grain characteristics that provide a viable and reliable indication of wear debris characteristics for machine condition monitoring. Yuan et al. [20] classify the debris through the abrasive grain boundary signal obtained by a new radial concave deviation (RCD) method.

**Figure 1:**
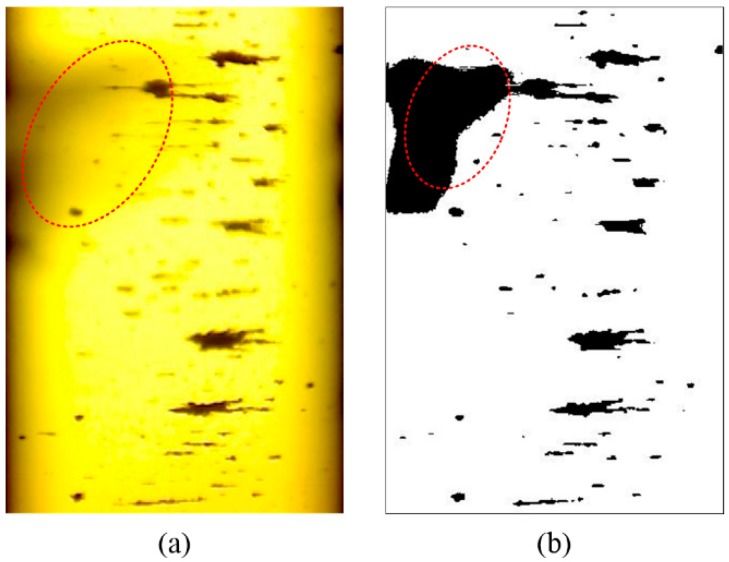
Effect of interference shadows on the binarization of on-line visual ferrograph (OLVF) ferrograms (shadow is encircled in red): (**a**) OLVF ferrogram; (**b**) results obtained by Otsu method.

The above methods are mostly proposed for off-line ferrograms, which are not applicable for the processing of OLVF ferrograms. In a complete wear monitoring process, the acquired number of OLVF ferrograms is very large. To facilitate OLVF ferrogram storage, OLVF ferrogram resolution is low, leading to difficulty in the extraction of wear debris characteristics. Meanwhile, to adapt to the online monitoring environment of the industrial site, the OLVF’s optical imaging system is simple and compact in structure such that the optical magnification (approximately 2 times) is considerably smaller than that of the ferroscopy microscope. Under this condition, the detailed information of the debris is limited, which leads to difficulty in extracting the particles’ features. In addition, a large number of bubbles will be generated under the action of valves, oil pumps, and gears during the lube oil cycle. As shown in Figure 1, when OLVF is used to perform online sampling analysis of the gearbox, the bubble flows through the probe, appearing as a dynamically changing interference shadow. It is difficult to eliminate the interference shadow from the OLVF ferrogram using the traditional Otsu binarization method. The interference shadow is misidentified as wear debris, resulting in large errors in the extracted wear debris characteristics.

In recent years, the curvelet transform has been of wide interest to domestic and foreign researchers because of its approximate optimal expression of line singularity features in images. The curvelet transform has been successfully applied to image denoising and fusion and has achieved good results [21]. The curvelet transform introduces directional parameters; therefore, it has better directivity than the two-dimensional wavelet transform. At the same time, the curvelet transform combines the advantages of the ridgelet transform in expressing linear features and the advantages of two-dimensional wavelets in expressing point features and is thus more suitable for multiscale analysis of images with high edge information. On this basis, the Fast Discrete Curvelet Transform (FDCT) was proposed by Candes et al. [22]. FDCT completely abandons the Ridgelet transform and directly gives the concrete expression of the Curvelet transform in the frequency domain. The image multiscale decomposition process by FDCT is as follows: the image is subjected to a fast Fourier transform; then, the frequency domain coefficients are resampled in different directions at different scales; finally, the fast Fourier transform is performed after the new coefficients are windowed, and the progeny coefficients of different scales in different directions can be obtained. Nguyen et al. [23] proposed Uniform Discrete Curvelet Transform (UDCT), the main idea of which was derived from FDCT and the filter bank-based Contourlet transform. Its implementation is mainly based on the FFT algorithm, but its curvelet function is designed in a multiscale filter bank structure. Compared with the Contourlet transform [24], UDCT has better frequency response, lower coefficient redundancy, and lower computational complexity, making it more suitable for engineering applications [25]. Feng et al. [26] used UDCT to analyze OLVF spectra at multi-scale. The nonlinear transformation is used to UDCT coefficients and achieve denoising of abrasive images, binary abrasive images under interference conditions were obtained, and has achieved good results.

## 2. Methods

### 2.1. Uniform Discrete Curvelet Transform

#### 2.1.1. UDCTs Window Function

**Figure 2:**
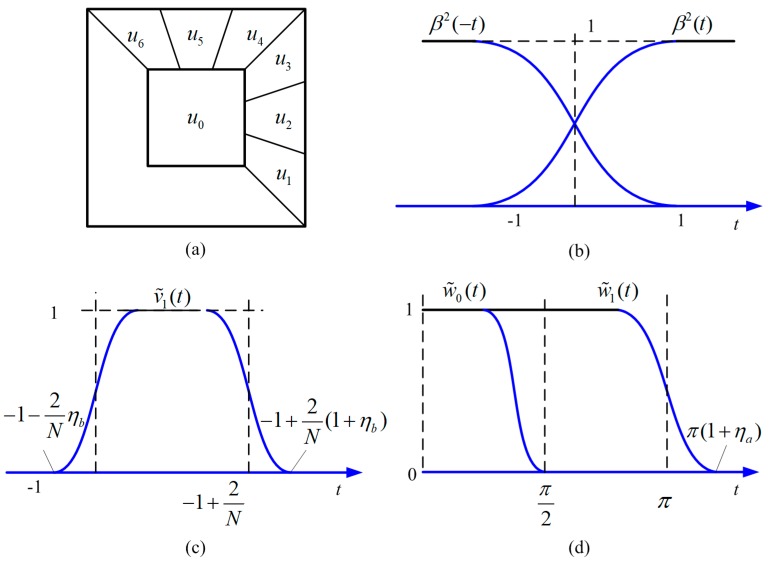
Structure of the Uniform Discrete Curvelet Transform (UDCT) window function: (**a**) Regions of essential support of $u_{l}(\omega )$ (N=3); (**b**) One-dimensional smooth projection function; (**c**) Low-pass window function and the bandpass window function; (**d**) Angle window function.

UDCT defines 2N+1 smooth curvelet window functions $u_{l}(\omega )$, l=0, 1,⋅⋅⋅,N to divide the frequency domain, where ω represents $\left(\omega_{1},\omega_{2}\right)$. These window functions meet the following criteria:

- All window functions are considered to have a 2π period in both the $\omega_{1}$ and $\omega_{2}$ directions, and the domain of $u_{l}(\omega )$ is ${\left[-\pi ,\pi \right]}^{2}$.
- As shown in Figure 2a, $u_{0}(\omega )$ is a square low-pass filter window with the support domain ${\left[-\pi /2,\pi /2\right]}^{2}$. Further, the support fields of the remaining 2N window functions are wedge-shaped.
- $u_{l}(\omega )$ is a smooth compact support function, and the central region function value is 1.
- $u_{0}(\omega )+\sum_{l=1}^{2N}\left[u_{l}(\omega )+u_{l}(\omega )\right]=1$.

To construct $u_{l}(\omega )$, a one-dimensional projection function β(t) was defined first, which smoothly transitioned from 0 to 1 in the range of [−1,1]; parameters $\eta_{a}$ and $\eta_{b}$ were introduced to control the transition band width of $u_{0}(\omega )$ and $u_{l}(\omega )$, respectively. $u_{0}(\omega )$ is obtained by multiplying two one-dimensional functions, and $u_{l}(\omega )$ is constructed by multiplying a square window function and 2N angular functions.

β(t) must meet the following requirements:

(1) $$\{\begin{array}{l} \beta \left(t\right)=1\;\;t\geq 1\\ \beta \left(t\right)=0\;\;t\leq -1\\ \beta^{2}\left(t\right)+\beta^{2}\left(-t\right)=1\;-1<t<1 \end{array}$$

As shown in Figure 2b, β(t) that satisfies the condition can be constructed as

(2) $$\beta^{2}\left(t\right)=-\frac{5}{32}t^{7}+\frac{21}{32}t^{5}-\frac{35}{32}t^{3}+\frac{35}{32}t+\frac{1}{2}\;t\in \left[-1,1\right]$$

Then, based on β(t), ${\tilde{w}}_{0}(t)$ and ${\tilde{w}}_{1}(t)$ are defined as

(3) $${\tilde{w}}_{1}(t)=\beta \left(\frac{\pi -\left|t\right|}{\pi \eta_{a}}\right)$$

(4) $${\tilde{w}}_{0}(t)={\tilde{w}}_{1}(2t(1+\eta_{a}))$$

where the support domain of ${\tilde{w}}_{0}(t)$ is [−π/2,π/2]. ${\tilde{w}}_{0}(t)$ and ${\tilde{w}}_{1}(t)$ are plotted in Figure 2d.

Thus, the expressions for the low-pass window function $w_{0}(\omega )$ and the bandpass window function $w_{1}(\omega )$ are defined as

(5) $$w_{0}(\omega )={\tilde{w}}_{0}(\omega_{1}){\tilde{w}}_{0}(\omega_{2})$$

(6) $$w_{1}(\omega )={\left(1-w_{0}^{2}(\omega )\right)}^{1/2}{\tilde{w}}_{1}(\omega_{1}){\tilde{w}}_{1}(\omega_{2})$$

Similarly, the angle window functions can be constructed as

(7) $$v_{1}(t)=\beta (\frac{2/N-1-t}{2\eta_{b}/N})\beta (\frac{t+1}{2\eta_{b}/N})$$

(8) $$v_{l}(t)=v_{1}(t)(t-2(l-1)/N)$$

The square low-pass filter window $u_{0}(\omega )$ is constructed to a periodic $w_{0}(\omega )$. Additionally, the 2N wedge-shaped curved window function $u_{l}(\omega )$ can be obtained by periodic $v_{l}(\omega )$ and $w_{1}(\omega )$.

#### 2.1.2. UDCT Frequency Domain Filter Bank

Suppose $N=k\cdot 2^{n}$, k>0, n≥0. A UDCT frequency domain filter bank was constructed by $u_{l}(\omega )$. 2N+1 filters are defined as

(9) $$\left\{ \begin{array}{l} F_{0}(\omega )=2u_{0}(\omega )\\ F_{l}(\omega )=2^{\frac{n+3}{2}}u_{l}(\omega )\\ G_{l}(\omega )=F_{l}(\omega ) \end{array}\right.,\;l=1,\cdot \cdot \cdot ,2N$$

where $F_{0}(\omega )$ is a low-pass filter, $F_{l}(\omega )$ is a directional filter, and $G_{l}(\omega )$ is a reconstruction filter that has the same form as the directional filter. The corresponding sampling matrix of the filter bank is

(10) $$\left\{ \begin{array}{l} D_{0}=diag\{2,2\},\;\;\;l=0\\ D_{1}=diag\{2,2^{n+1}\},\;\;l=1,\cdot \cdot \cdot ,N\\ D_{2}=diag\{2^{n+1},2\},\;\;l=N+1,\cdot \cdot \cdot ,2N \end{array}\right.$$

**Figure 3:**
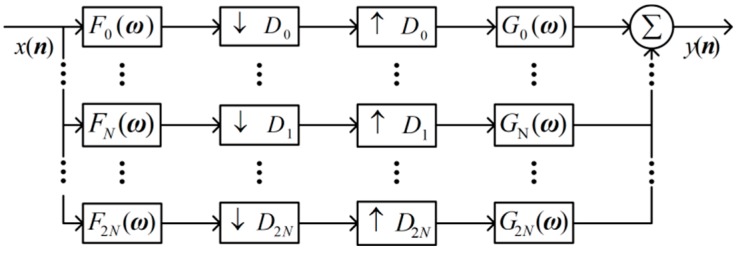
Structure of the UDCT filter bank.

The specific structure of the UDCT filter bank is shown in Figure 3. 2D signal x(n) is first filtered by $F_{l}(\omega )$, and the subband coefficients are obtained by downsampling. At the synthesis side, the subband coefficients are first upsampled, then convolved with $G_{l}(\omega )$. Finally, the reconstructed output signal is obtained by $y(n)=Real(\sum_{i=0}^{2N}x_{l}(\omega ))$. To avoid frequency aliasing during down sampling, the window function parameters $\eta_{a}$ and $\eta_{b}$ should meet

(11) $$\left\{ \begin{array}{l} 0<\eta_{a}\leq (\sqrt{17}-3)/4\\ 0<\eta_{b}\leq 0.5\\ 2(1+\eta_{a})(1+2\eta_{b})\leq k \end{array}\right.$$

### 2.2. Multiscale Decomposition of OLVF Ferrograms Based on UDCT

**Figure 4:**
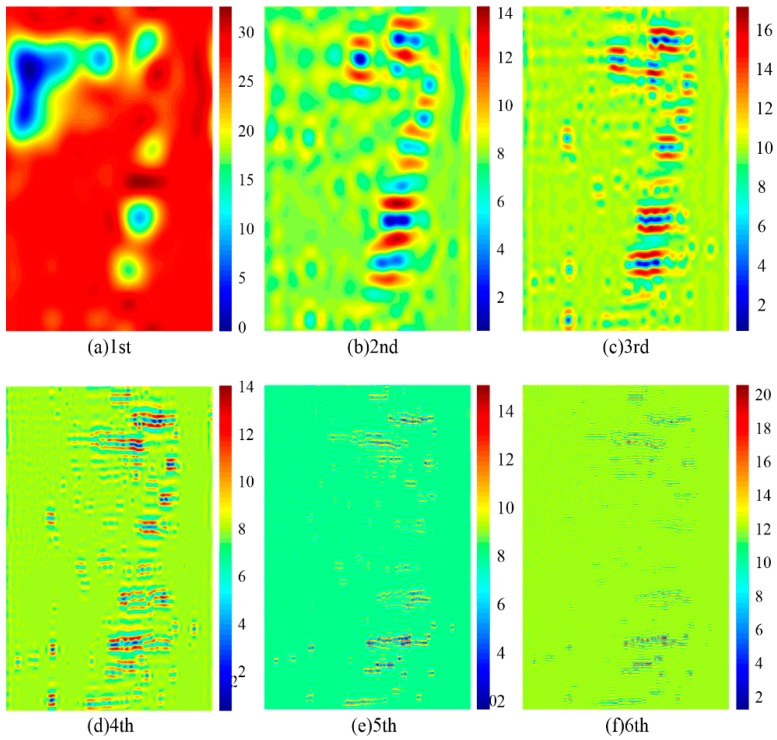
Contour single-layer coefficients from 1 to 6: (**a**) the first layer; (**b**) the second layer; (**c**) the third layer; (**d**) the fourth layer; (**e**) the fifth layer; (**f**) the sixth layer.

Multiscale and multidirectional decomposition of the OLVF ferrogram was performed using UDCT. Compared with image wavelet decomposition, UDCT not only has vertical, horizontal and diagonal information but also has richer directional information. The OLVF ferrogram was decomposed by UDCT into a low-frequency part and a mid-high-frequency part. UDCT energy is mainly concentrated on the low-frequency coefficient that reflects the overview of the ferrogram. The mid-high-frequency coefficients mainly reflect the multidirectional edge feature information of the wear debris in the ferrogram. After gray processing of the ferrogram, shown in Figure 1, a six-layer UDCT was performed. Only the coefficient of the UDCT single-layer was retained, while the coefficients of the remaining layers were set to zero. The image was reconstructed to obtain a gray contour map of the reconstructed ferrogram, as shown in Figure 4. Obviously, the interference shadow is a low-frequency signal whose energy is mainly concentrated in low-frequency UDCT coefficients.

**Figure 5:**
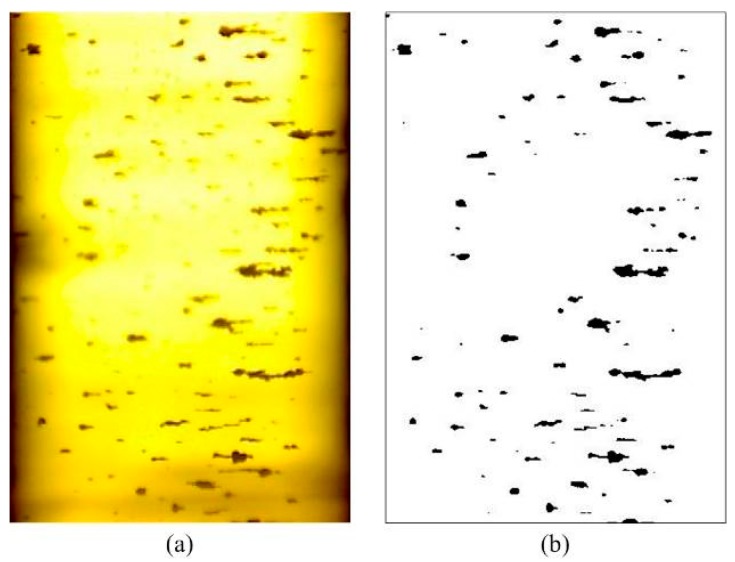
Results after removing low-frequency UDCT coefficients from a ferrogram with small wear debris. (**a**) on-line visual ferrograph (OLVF) ferrogram; (**b**) removing low-frequency coefficients after UDCT decomposition.

**Figure 6:**
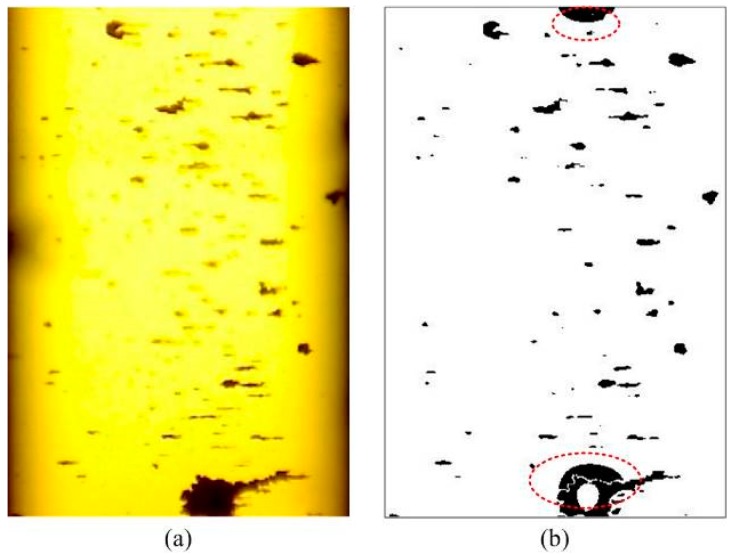
Results after removing low-frequency UDCT coefficients from a ferrogram with large wear debris (red circles show wrongly detected particles). (**a**) Large-size debris in the ferrogram; (**b**) erroneous binarization results.

As shown in Figure 5, by removing the low-frequency coefficients, interference shadow in the ferrogram with small wear debris can be effectively removed. However, when there are large-sized debris in the ferrogram (Figure 6a), the gray value of the area covered by large debris changes only slightly, and the energy of the large debris after UDCT decomposition is mainly decomposed to low-frequency coefficients. This may be confused with low-frequency interference shadows. At this point, holes may be generated in the middle of large debris due to energy loss after removing the low-frequency coefficients, and erroneous binarization results are obtained (Figure 6b). When the size of the wear debris is small in the ferrogram, the wear debris image contains little low-frequency information. At this point, the interference shadow can be effectively removed by removing the low-frequency coefficient reconstructed ferrogram after UDCT decomposing without affecting the image characteristics of wear debris. However, when the size of the wear debris in the ferrogram is large, the wear debris image contains considerable low-frequency information. Consequently, the interference shadow cannot be eliminated by removing the low-frequency coefficient reconstructed ferrogram after UDCT decomposition due to a great loss of image characteristics of wear debris. Therefore low-frequency coefficient should be suppressed and transformed to a certain extent in order to achieve good interference shadow removal.

### 2.3. Nonlinear Enhancement of OLVF Ferrogram Based on UDCT

A nonlinear function was used to process the low-frequency coefficients of the UDCT transform to eliminate the interference shadow information. The nonlinear function [27] is defined as follows:

(12) $$f(x)=\frac{sigm\left[c(x-b)\right]-sigm\left[-c(x+b)\right]}{sigm\left[c(1-b)\right]-sigm\left[-c(1+b)\right]}$$

whereby sigm(x)=1/[1+exp(−x)]; 0<b<1, b is the input fragmentation threshold and c is the control coefficient of the enhanced data rate.

#### 2.3.1. High-Frequency Denoising

High-frequency sub-band coefficients reflect the details and edge features of OLVF ferrograms and contain noise. The coefficients for UDCT were adjusted to achieve denoise processing of the OLVF ferrogram. The piecewise nonlinear adjustment function is

(13) $$y=\left\{ \begin{array}{l} s\cdot f(x),\;\;x\geq T\\ 0,\;x<T \end{array}\right.$$

where s is an adjustable coefficient; x=|C(i,j)|/Max; the noise evaluation parameter σ=median(|C(i,j)|/0.6745); the threshold T=λ·σ; the coefficient λ=3~4; the adjusted high frequency progeny coefficient is $C_{Hnew}(i,j)=Max\cdot y$.

#### 2.3.2. Low-Frequency Suppression

The interference shadows are found in the low frequencies, and the processing of low-frequency coefficients is the key. To suppress interference shadows, low-frequency progeny coefficients need to be suppressed. A piecewise nonlinear function was used to process the low-frequency progeny coefficients.

(14) $$y=\left\{ \begin{array}{l} s_{1}\cdot f(x,c_{1}),\;\;x\geq b\\ s_{2}\cdot f(x,c_{2}),\;\;x<b \end{array}\right.$$

where $s_{1}$ and $s_{2}$ are adjustable coefficients; x=|C(i,j)|/Max; $b=(\sum_{i=1}^{M}\sum_{j=1}^{N}\left|C(i,j)\right|)/(M\cdot N\cdot Max)$; C(i,j) are the coefficients obtained by UDCT; Max is the largest absolute value for C(i,j); and M and N are the matrix dimension. To ensure continuity of the piecewise function, $s_{2}=s_{1}\times f\left(b,c_{1}\right)/f\left(b,c_{2}\right)$. Then, the adjusted low frequency progeny coefficient is $C_{Lnew}(i,j)=Max\cdot y$.

The steps of the UDCT-based OLVF spectrum anti-interference binarization algorithm are as follows:

1. The background subtraction method is used to subtract the OLVF spectrum from the background spectrum, and grayscaled to obtain an OLVF spectrum that eliminates background interference;
2. The method of uniform discrete curvelet transform is performed on the OLVF spectrum to obtain a series of high-frequency and low-frequency progeny coefficients;
3. The nonlinear transformation of low-frequency progeny coefficients is segmented, and the low-frequency interference shadow energy is suppressed; then, the high-frequency progeny index is subjected to threshold denoising, with the remaining progeny coefficients unchanged;
4. The progeny coefficients are integrated to perform the inverse discrete curvelet inverse transform to obtain the OLVF spectrum after suppressing the interference shadow;
5. A binarization process is used on the processed spectral slice OLVF with automatic threshold iterative method.

**Figure 7:**
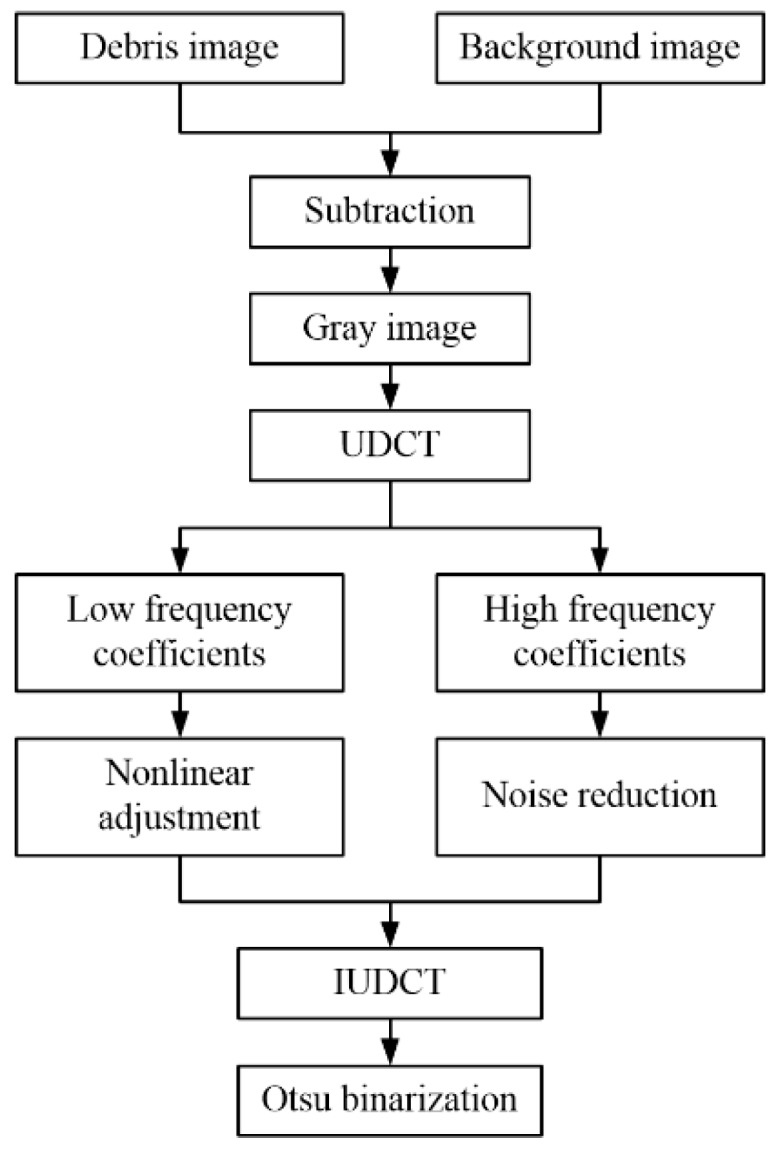
Spectral binarization based on uniform discrete curvelet transform.

The specific algorithm flow is shown in Figure 7.

## 3. Comparison with Other Methods

**Figure 8:**
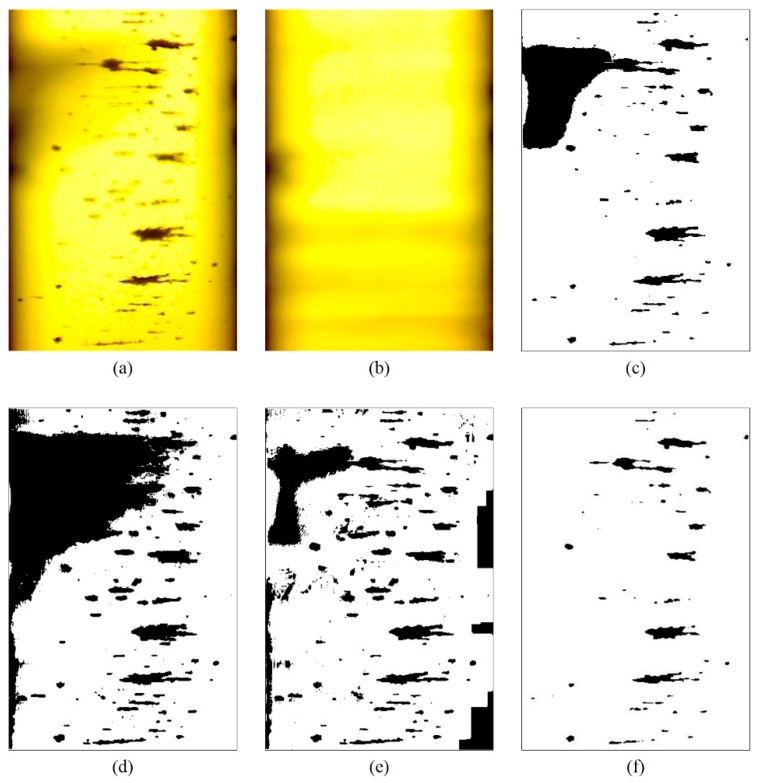
Comparison of the processing results of different binarization methods for a high-interference ferrogram. (**a**) Wear debris image of Figure 1a; (**b**) Background picture; (**c**) Otsu method; (**d**) Kittler method; (**e**) Niblack method; (**f**) the proposed method.

As shown in Figure 8, the proposed method was compared with three commonly used binarization methods: the Otsu method, the Kittler method, and the Niblack method. The calculation parameters of the proposed method are $c_{1}=5$, $s_{1}=0.45$, and $c_{2}=1$. As shown in Figure 1, the OLVF ferrogram has dark gray cloud-like interference shadows. The Otsu method can identify most of the wear debris in the ferrogram but also misidentifies the interference shadow at the bottom as wear debris. The Kittler method has good ability to identify low-contrast image objects. As a result, the part with dark gray shadows in the ferrogram is almost completely misidentified as wear debris. The Niblack method caused an intumescent effect on the ferrogram. The morphology of the wear debris binarized by the Niblack method is different from the actual one. The local interference shadows at the bottom of the ferrogram are misidentified as wear debris. Figure 8d shows the results obtained by the proposed method. The dark gray interference shadow is effectively removed, the edge of the wear debris image is clear, and the shape of the wear debris is similar to the actual shape.

## 4. Application in Wear Monitoring of Gearbox

**Figure 9:**
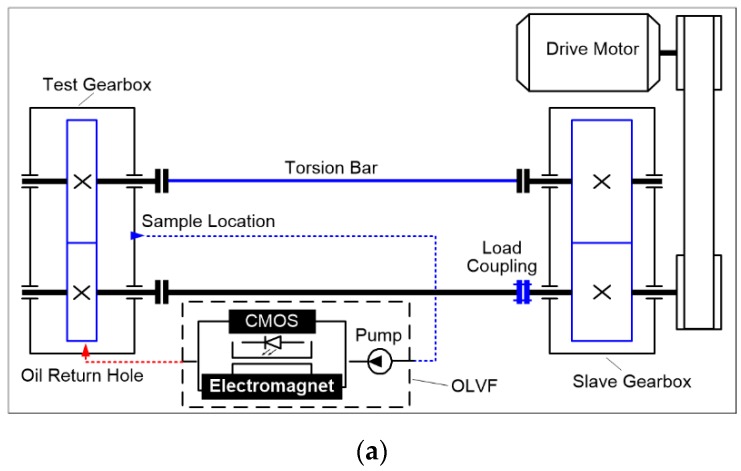

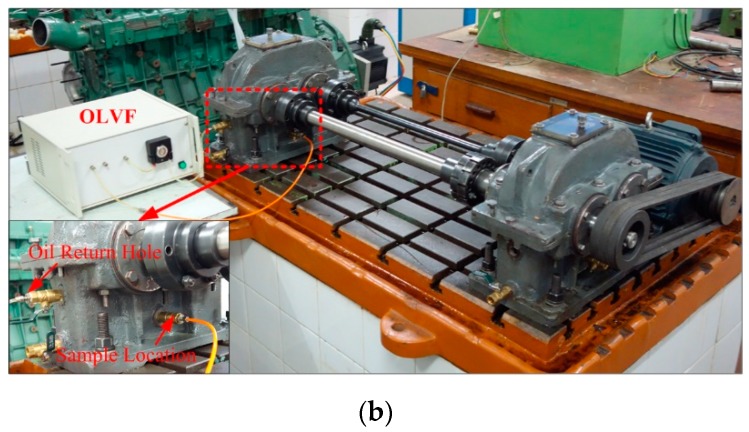
Back-to-back gear test rig: (**a**) schematic; (**b**) photograph.

A gear wear experiment was conducted in a back-to-back setup, shown in Figure 9. The OLVF was used to monitor gear wear in real time. The test was carried out for 51.87 h. A total of 1557 OLVF ferrograms were obtained during the test.

**Figure 10:**
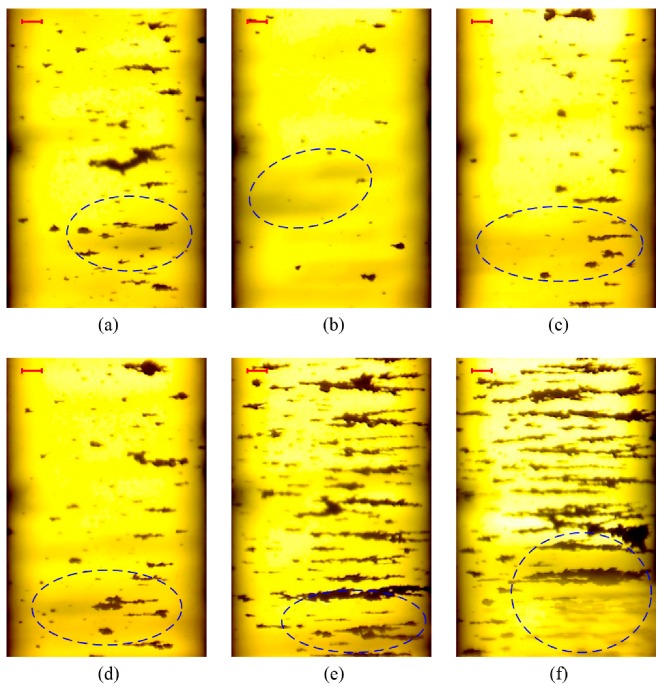
Representative ferrograms with interference shadows (the bar is 100 μm): (**a**) 22 min; (**b**) 2 h 26 min; (**c**) 4 h 6 min; (**d**) 5 h 8 min; (**e**) 16 h 6 min; (**f**) 21 h 50 min.

**Figure 11:**
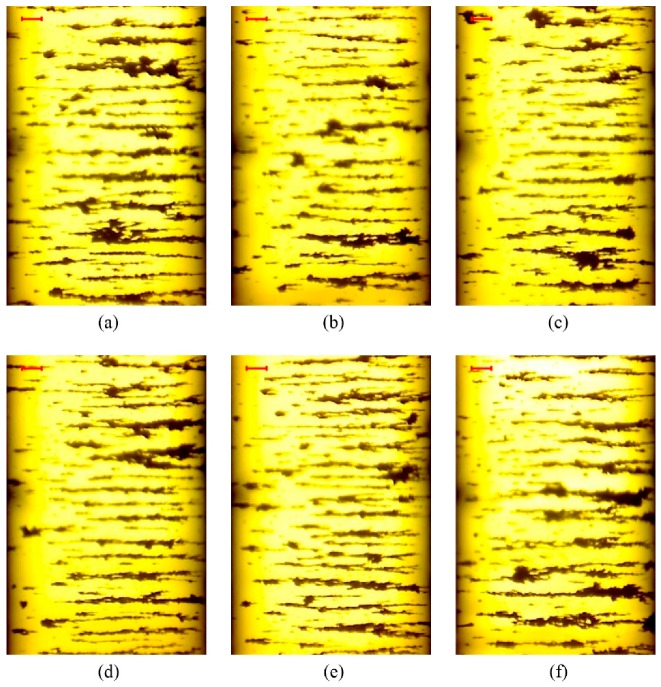
Representative ferrograms without interference shadows (the bar is 100 μm): (**a**) 25 h; (**b**) 30 h; (**c**) 35 h; (**d**) 40 h; (**e**) 45 h; (**f**) 50 h.

**Figure 12:**
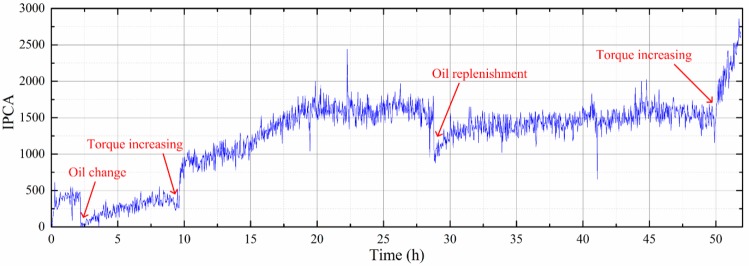
Variations of index of particle coverage area (IPCA) from the proposed method.

**Figure 13:**
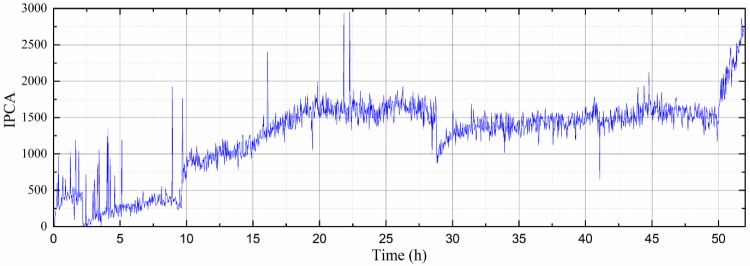
Variations of IPCA from the Otsu method.

**Figure 14:**
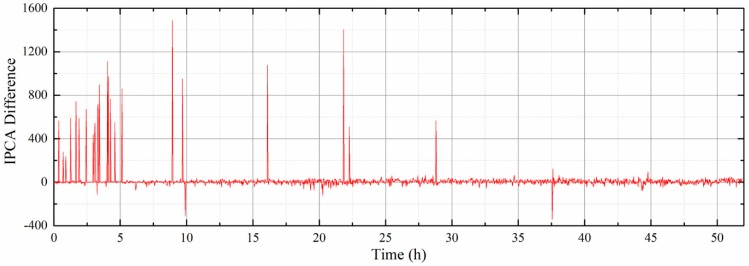
IPCA difference of two methods.

Figure 10 shows representative OLVF ferrograms with bubble interference. Figure 11 shows typical OLVF ferrograms after 25 h. The OLVF ferrograms were segmented by the proposed method and the Otsu method, respectively. And the index of particle coverage area (IPCA) [26] was calculated by binarized OLVF ferrograms, and the variation of the IPCA curve was obtained, as shown in Figure 12 and Figure 13. During the first five hours of the experiment, a large amount of bubbles was present in the lube oil due to the gear agitation. At this time, using the Otsu method to extract the IPCA values will produce large errors. There are many glitch impulses on the IPCA curve, as shown in Figure 13. Figure 14 is an image obtained by subtracting IPCA curves of the two methods. During the first five hours of the experiment, there have a great difference with the IPCA values extracted by this method and Otsu method. After about 30 h of experimentation, there is basically no difference in the experimental results of the two methods. Comparatively, the proposed method can better suppress the bubble interference existing in the OLVF ferrograms and reduce the IPCA glitch impulses generated by the bubbles, as shown in Figure 12. The bubbles in the lube oil are gradually reduced after 5 h, and both methods can obtain a good IPCA curve.

Obviously, IPCA has more glitch impulses before 5 h. The reason for this is because at the beginning of the experiment, bubbles are easily generated due to gear agitation because of the viscous lube oil. As the experiment progressed, the temperature of the lube oil increased and the viscosity decreased, and the generated bubbles were reduced, so that the interference of the bubbles on the OLVF spectrum was reduced.

## 5. Conclusions

Multiscale analysis of OLVF ferrograms was performed using UDCT. Bubbles appear as a low frequency interference shadow in OLVF ferrograms. The proposed anti-interference segmentation method for the OLVF ferrograms was used to suppress the low-frequency coefficients of UDCT to eliminate interference shadow in the ferrograms. A wear debris image with a clear edge can be obtained. Additionally, the method is used in online gear wear monitoring. Additionally, the initial stage IPCA glitch impulses caused by bubbles can be effectively suppressed or eliminated. The proposed method can meet the requirements of OLVF online sampling analysis, which provides more accurate data for the subsequent extraction of wear debris characteristics.
